# Opium Smoking

**Published:** 1883-08

**Authors:** 


### OPIUM SMOKING.

NEITHER Shy Lee nor any Chinaman will admit that opium sends him to sleep. "I no go to sleepee," he says in his low, musical way. "I sometimes smokee till I no can sleepee. Then I walkee about, and then I smokee again." His grave, still manner suggests that he lives somewhere nearly midway between the world of reality and the land of dreams. Even while he is speaking, he retires to a small, dark room at the back of his shop, and sinks down with his legs crossed, on a low bed. The appliances for opium smoking are already there, just as he has left them some time before. They consist of a wooden tray, a lamp, a small receptacle for opium, a piece of strong wire, and a pipe. The latter is a cumbrous implement, somewhat resembling a flute, with a cup-shaped piece of wood fitted into the mouthpiece. The lamp is diminutive, and is covered by a sort of inverted glass tumbler with a small round hole at the base. Resting his head upon a pillow, Shy Lee draws the tray toward him and begins to prepare his pipe. The process is long and tedious, but apparently productive of a deep and solemn delight. Shy Lee proceeds slowly, carefully, and with a solemn smile. A little of the opium is taken up on the end of the piece of wire and held over the lamp, where it half burns and half boils, as if it were resin. Then a little more of opium is taken up and similarly treated. When it is burned sufficiently, he rolls it about on the bowl of his pipe, still attached to the wire, until it has been converted into a small cone. This he inserts into a perforation in the wooden cup already mentioned, withdraws the wire, and flattens the opium with his finger. He is now ready to smoke, and, holding the bowl of his pipe over the light of the lamp, he sucks at the mouthpiece with a vigor unknown to ordinary smokers, at the same time expelling the smoke through his nostrils. Twenty seconds or so, and he again goes through the same labor of preparation, with the same smile of grave enjoyment. As he consumes one little cone of opium after another a film comes over his eyes, and he stares with set features; but he is still sufficiently conscious to be aware that some courtesy is demanded of him, and so he dreamily prepares another pipe and hands it to his guest.

---

ANOTHER scandal exploded: "They say Smith and his wife do not get along well together; is that true!" asked Brown. "Not a bit of it," replied Fogg; "they're never together."—*Boston Transcript.*

FEAR OF DISEASE.—It is said that while the plague was raging in Buenos Ayres, the grave-diggers bore charmed lives. Of the three hundred men so employed, not one died of the disease. It has often been noticed that during the prevalence of pestilential diseases, physicians, undertakers, nurses and grave-diggers, whose business compelled constant liability to infection, have usually escaped in a far greater ratio than their numbers would warrant. The "charm" of this immunity from the prevailing scourge is very simple. They are not *scared.* They are *positive* to the disease and repel its attack. *Fear* is a great ally of death. Whoever is afraid of disease is in a *negative* condition, and really invites its approach. And thus it is the world over. The brave die but once, while cowards die many times. Much unnecessary alarm exists in every community in regard to many diseases. We are, it is true, all liable to sickness and death. But if we are all sober, cleanly and brave of heart, we need have no fear of disease of body or mind.—*Golden Rule.*

